# Preparation of recombinant *Kluyveromyces lactis* agents for simultaneous degradation of two mycotoxins

**DOI:** 10.1186/s13568-022-01361-6

**Published:** 2022-02-18

**Authors:** Yu Xia, Yangyu Qiu, Zifeng Wu, Qianqian Cheng, Xiuyu Hu, Xiaobing Cui, Zhouping Wang

**Affiliations:** 1 State Key Laboratory of Food Science and Technology, Jiangnan University, Wuxi, 214122 China; 2 School of Food Science and Technology, Jiangnan University, Wuxi, 214122 China; 3 China Biotech Fermentation Industry Association, Beijing, 100 833 China; 4 Anhui Heiwa Food-Jiangnan University Joint R&D Center, Anhui Heiwa Food Technology Co. LTD, Bozhou, 233600 China

**Keywords:** *Kluyveromyces lactis*, Yeast agent preparation, Aflatoxin B_1_, Zearalenone, Degradation

## Abstract

Aflatoxin B_1_ (AFB_1_) and zearalenone (ZEN) are widely distributed in corns, peanuts, and other cereals, causing serious threat to food safety and human health. As shown by our previous studies, the recombinant yeast strain *Kluyveromyces lactis* GG799(pKLAC1-ZPF1) had the ability of degrading AFB_1_ and ZEN simultaneously. In this work, the agent preparation process was optimized for *K. lactis* GG799(pKLAC1-ZPF1), and the storage conditions of the prepared yeast agents were investigated, for obtaining the products with high storage activities and potent mycotoxin degradation efficiency. The optimal preparation process was as follows: centrifugation at 6000 rpm for 15 min for collection of the yeast cells, spray drying with the ratio of protective compounds to yeast cells at 3:1 (w/w) and then stored at − 20 °C. Simultaneous degradation tests of AFB_1_ and ZEN were performed using the supernatants of reactivated yeast agents after three months of storage, and the degradation ratios for AFB_1_ and ZEN in reaction system 1 (70.0 mmol/L malonic buffer, pH 4.5, with 1.0 mmol/L MnSO_4_, 0.1 mmol/L H_2_O_2_, 5.0 μg/mL AFB_1_ and ZEN, respectively) were 48.2 ± 3.2% and 34.8 ± 2.8%, while that for ZEN in reaction system 2 (50.0 mmol/L Tris–HCl, pH 7.5, with 5.0 μg/mL AFB_1_ and ZEN, respectively) was 30.1 ± 2.7%. Besides, the supernatants of reactivated yeast agents degraded more than 80% of AFB_1_ and 55% of ZEN in contaminated peanuts after twice treatments. Results of this work suggested that the optimized process for *K. lactis* GG799(pKLAC1-ZPF1) was with high value for industrial applications.

## Key points

- The recombinant strain *K. lactis* GG799(pKLAC1-ZPF1) was food-grade and safe for food and feed industry.
- The preparation processes of the *K. lactis* GG799(pKLAC1-ZPF1) agents were optimized, and the storage properties were investigated.
- The culture supernatants of the reactivated yeast agents, rather than the purified enzymes, were used to degrade AFB_1_ and ZEN in practical samples for reducing the production cost.

## Introduction

Mycotoxins are secondary metabolites produced by fungi which could lead to adverse effects after intake by human beings or animals(Ayofemi Olalekan Adeyeye 2020). Aflatoxin B_1_ (AFB_1_) and zearalenone (ZEN), as two major fungal toxins, are widely distributed in food stuffs such as peanuts, corns, and other grains, causing serious damage to human or animal health (Lee and Ryu 2017; Nazhand et al. 2020; Eskola et al. 2020). Currently, the main detoxification methods for mycotoxins include physical, chemical, and biological ones. However, the physical methods including heat treatment, adsorption, separation and irradiation, tend to destroy flavor and nutrients of grains and cannot completely reduce mycotoxins (Wu et al. 2021). Chemical methods involve ozone detoxification, hydrogen peroxide treatment, alkali treatment, sodium carbonate soaking treatment and ammoniation (Rempe et al. 2013). Although chemical methods can alter the structure of mycotoxins and detoxify mycotoxins from contaminated cereals, their usage can also easily cause secondary pollution (Xu et al. 2021). Meanwhile, the high risk and low efficiency limit the application of chemical methods in food industry (Ryu et al. 1999; Loi et al. 2017). Biological methods are effective for degradation of fungal toxins gently and efficiently (Adebo et al. 2017; Wang et al. 2019b). Currently, several enzymes have been identified having the ability for degradation of AFB_1_ and ZEN respectively, and most of them have been expressed recombinantly in *Escherichia coli* and yeast systems. Considering the wide range and diversity of mycotoxins worldwide, there is an urgent demand for methods that can detoxify two or more mycotoxins simultaneously. *Kluyveromyces lactis* has emerged as one of the most prominent yeast species for research, and has been extensively used in food and feed industry for a long time due to their generally regarded as safe (GRAS) status (Spohner et al. 2016; van den Dungen et al. 2021). As a result, the recombinant strain *K. lactis* GG799(pKLAC1-ZPF1) was a food-safe strain. In our previous works, we selected zearalenone hydrolase ZHD101.1 (ZHD101 mutant V153H) from *Clonostachys rosea* and manganese peroxidase PhcMnp from *Phanerochaete chrysosporium* for construction of the fusion enzyme ZPF1. The enzyme ZHD101.1 specifically hydrolyzes ZEN into a nontoxic alkyl resorcinol product by destroying its lactone bond, while the enzyme PhcMnp oxidizes AFB_1_ to AFB_1_-8,9-dihydrodiol due to its high redox potential. Its oxidation is mainly through free radicals, which are produced by interaction of oxidized Mn^3+^ with dicarboxylic acid malonate. Besides, our previous studies showed that the recombinant yeast strain *K. lactis* GG799(pKLAC1-ZPF1) successfully achieved simultaneous degradation of AFB_1_ and ZEN (Xia et al. 2021a).

Yeast agents, which have no toxins creation, no harmful and residual effects, are excellent alternatives to antibiotics and have great potentials for application in feed industry (Zhong et al. 2018). Present research on the degradation of mycotoxins was mainly focused on the construction of recombinant strains, the optimization of cultural conditions and degradation reaction parameters (Jiang et al. 2008; Xiang et al. 2016). To satisfy the industrial production, the research on the preparation of agent using the described *K. lactis* GG799(pKLAC1-ZPF1) became urgently needed. In the process of yeast agents preparation, the centrifugation conditions after cultivation, the kind and the ratio of protective compounds, the drying process and treatment time would have impacts on the activity of the dried powders (Palmfeldt et al. 2003; Wang et al. 2019a). In addition, storage conditions are also important factors that should be taken into consideration for yeast agents preparation, in order to remain the microbial active (Sullivan and Bradford 2011). In general, selecting the appropriate preparation process can keep the viability of the cells at maximum extent, enhancing the expression levels and achieving high degradation efficiency of the mycotoxins. In this short communication, the agent preparation process was optimized for *K. lactis* GG799(pKLAC1-ZPF1), which provided an effective reference for toxin degradation in the field of grain feed industry and the industrial production of *K. lactis* GG799(pKLAC1-ZPF1) agents.

## Meterials and methods

### Strains, cultural conditions and chemicals

The recombinant strain *K. lactis* GG799(pKLAC1-ZPF1) (Xia et al. 2021a) were preserved in this laboratory. Recombinant strain was grown at 30 °C in YEPD medium (yeast extract 1%, peptone 2%, glucose 2%, pH 6.3). For induction expression of ZPF1, YEPG medium (1.0% yeast extract, 2.0% peptone, 2.0% galactose, 0.5 mmol/L MnSO_4_, 0.5 mmol/L hemin, pH 5.9) was used and the strain was cultivated at 30 °C, 200 rpm for 96 h.

AFB_1_ and ZEN were purchased from Pribolab (Shandong, China). In this research work, these standards were made into a 1.0 mg/mL stock solution with methanol or acetonitrile solvent and were stored in darkness at − 20 °C. Methanol, formic acid, and acetonitrile were purchased from Tedia (OH, USA). All other reagents and chemicals were of analytical reagent grade.

### Preparation of the *K. lactis* GG799(pKLAC1-ZPF1) agents

**Fig. 1:**
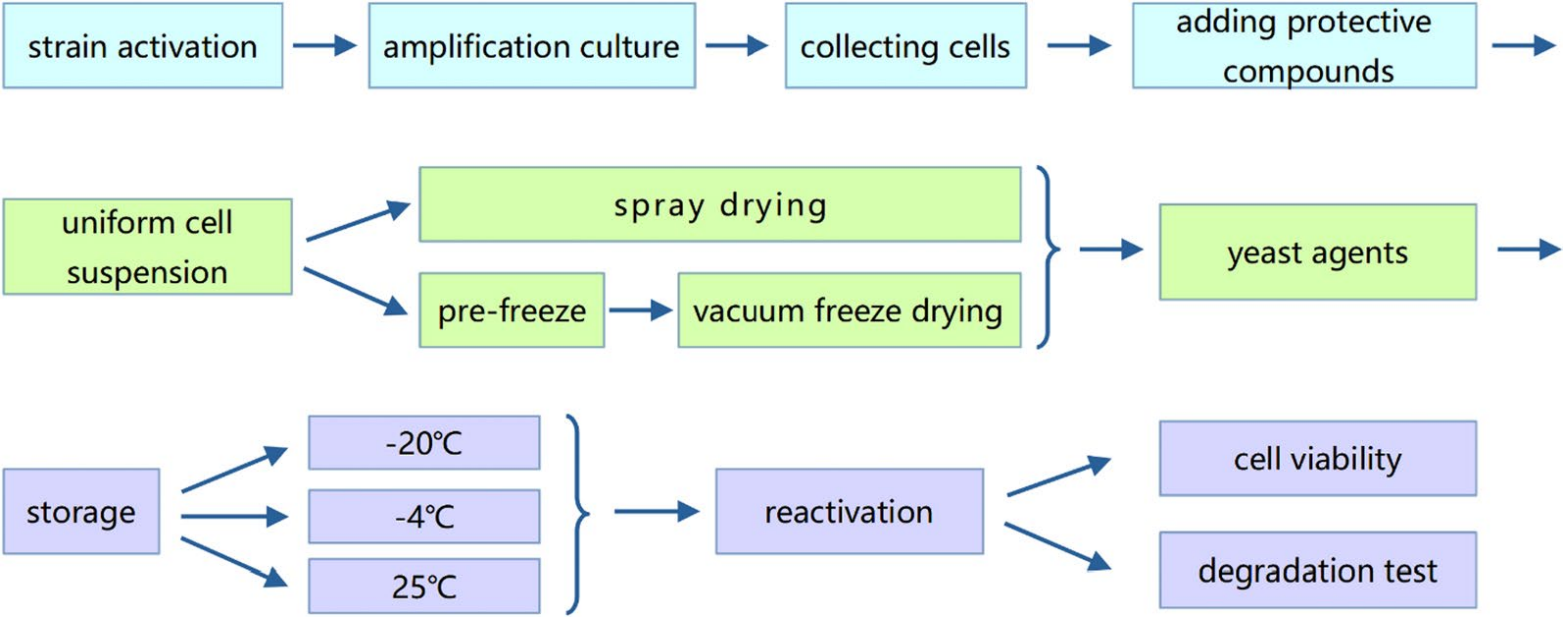
The process flow-chart for preparation of *K. lactis* GG799(pKLAC1-ZPF1) agents

The recombinant *K. lactis* GG799(pKLAC1-ZPF1) stored at − 80 °C was cultivated in the YEPD agar medium at 30 °C and incubated for 3 to 5 days for activation. Single colony was picked and cultivated in YEPD liquid medium at 30 °C, 200 rpm for 18 to 22 h. When the OD_600_ of the cultures reached 1.0, the seed solution was inoculated in a 5 L fermenter containing 2.0 L YEPD medium at 28 °C and aeration of 1.5 vvm for 35 h. The fermentation process was controlled at 28 °C, 100% oxygen solubility, pH 5.0. The cultures were centrifuged under different centrifugation conditions to collect the yeast cells. The yeast cells and the composite lyophilized protective compounds were mixed in different ratios and resuspended separately to prepare uniform cell suspensions. The suspensions were dried by different processes for preparation of *K. lactis* GG799(pKLAC1-ZPF1) agents (Fig. 1).

### Optimization of the *K. lactis* GG799(pKLAC1-ZPF1) agents process

The initial viable count of the yeast cells was measured by dilution coating with 50.0 mL of the culture. For the centrifugation conditions, the yeast cells were collected at 5000 to 7000 rpm, and the samples treated with each speed conditions were collected at 10 min, 15 min and 20 min, respectively. The centrifuged yeast cells were resuspended in physiological saline and the optimal centrifugation conditions were characterized by the number of viable yeast cells using the dilution coating method.

Different protective compounds protect the cells by different mechanisms, therefore, the protective compounds will be used in combination rather than singly in the actual production process. Thus, skimmed milk powder and maltose were chosen as the compound lyophilization protectant to provide protection and avoid damage to the cells during drying process (Han et al. 2019). The complex lyophilization protective compounds were mixed with yeast cells in different ratios at 1:1 (w/w), 1:2 (w/w), 1:3 (w/w) and 1:4 (w/w).

The cell suspension was prepared according to the above methods. A part of the obtained suspension was used for spray drying. The conditions for spray drying were as following (Stefanello et al. 2019): inlet air temperature was 135 °C, outlet air temperature was 60 °C, and solids content ranged from 15 to 25%. The other part was pre-frozen at − 80 °C for 3 h and then used for vacuum freeze drying. The conditions for vacuum freeze drying were as following (Xia et al. 2021b): temperature of condenser was -40 °C, degree of vacuum was 10 Pa, and drying time was 24 h. The dried samples were weighed and added to physiological saline, placed in an incubator at 30 °C for 30 min, and then diluted and coated. The optimal mixing ratio and drying process were determined according to the number of viable yeast cells.

### Storage of the *K. lactis* GG799(pKLAC1-ZPF1) agents and degradation test of AFB_1_ and ZEN

The *K. lactis* GG799(pKLAC1-ZPF1) agents were placed in sealed bags and stored at − 20 °C, − 4 °C and 25 °C, respectively. The appropriate amount of yeast agents was taken every month, and the viable yeast cells were counted by dilution coating after reactivation with 10.0 mL physiological saline.

The reactivated recombinant yeast was cultivated in YEPG medium containing 0.5 mmol/L MnSO_4_ and 0.5 mmol/L hemin at 30 °C for about 96 h at a speed of 200 rpm. The fermentation supernatants were collected by centrifugation at 8000 rpm and the protein samples were concentrated with 10.0 kDa ultrafiltration tubes. The degradation tests of AFB_1_ and ZEN were performed by the supernatants. Due to the two domains on the fusion enzyme ZPF1 (ZHD101.1 and PhcMnp) had different reaction requirements, the degradation efficiency of ZPF1 on AFB_1_ and ZEN was tested by two different reaction systems (Xia et al. 2021a). The reaction system 1 contained 70.0 mmol/L malonic acid buffer (pH 4.5) with 1.0 mmol/L MnSO_4_, 0.1 mmol/L H_2_O_2_, 1.0 mg/mL culture supernatant protein, 5.0 µg/mL AFB_1_ and 5.0 µg/mL ZEN (Wang et al. 2019b), while the reaction system 2 contained 50.0 mmol/L Tris–HCl (pH 7.5) with 5.0 µg/mL AFB_1_ and 5.0 µg/mL ZEN (Xiang et al. 2016).

To degrade the two mycotoxins in reaction system 1, the solution containing all samples was incubated at 30 °C for 9 h and then 3.0 mL of methanol was added to terminate the reaction (Wang et al. 2019b). To degrade the two mycotoxins in reaction system 2, the solution containing all samples was incubated at 37 °C for 30 min and then 1.0 mL of methanol was added to terminate the reaction (Xiang et al. 2016) The degradation ratios of ZEN in reaction systems 1 and 2 were denoted as ZEN-1 and ZEN-2, respectively.

### Simultaneous degradation of AFB_1_ and ZEN in contaminated peanut samples

The peanut samples were shelled to remove the impurities and dust (Yang et al. 2020). The samples were prepared into a powder with a particle size less than 2.0 mm by a homogenizer. The powder samples (5.0 g) mixed with 10.0 mL water were stirred thoroughly, and then autoclaved at 121 °C for 20 min. The AFB_1_ standards and ZEN standards were added to the peanut samples at different concentrations respectively (100.0 μg/kg, 500.0 μg/kg, 2000.0 μg/kg) and the samples were thoroughly stirred. Then the samples were mixed with the supernatants from the reactivated and cultured yeast agents. The final volume of the reaction system was 35.0 mL containing 70.0 mmol/L malonic acid buffer (pH 4.5) with 1.0 mmol/L MnSO_4_, 0.1 mmol/L H_2_O_2_, 1.0 mg/mL culture supernatant protein. The concentration of AFB_1_ and ZEN was described above. The reaction was performed at 37 °C for 40 h. After the first degradation treatment, the liquid phase and the solid phase in the samples were separated, and the same volume of the supernatants were added to these solid phase samples again, for the twice treatments of the mycotoxins with the methods described above. The samples were analyzed by UPLC-MS for detection of the residual concentration of AFB_1_ and ZEN.

### Quantitative assay of AFB_1_ and ZEN

The residual degradation samples containing the mycotoxins were filtered through a 0.22 µm filter and the residual concentrations of AFB_1_ and ZEN were detected by UPLC-MS. The specific operating parameters were as following: chromatographic was performed using ACQUITY UPLC® BEH C18 (2.1 × 50 mm, 1.7 µm particle size); the mobile phase was acetonitrile/ water/formic acid; column temperature was 40 °C; flow rate was 0.3 mL/min. Mass spectrometry parameters were as following: electrospray ion source; multiple reaction monitoring modes (MRM); heating gas temperature was 500 °C; cone Voltage was 3.0 kV; ion source temperature was 150 °C; desolventizing gas rate was 800 L/h (Xia et al. 2021a).

## Results

### Effects of centrifugal conditions on the cell viability of the recombinant strain

**Fig. 2:**
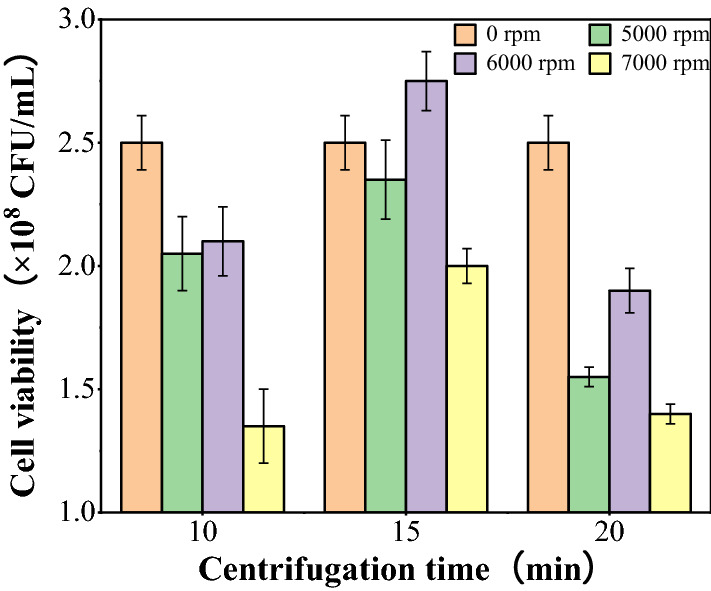
The cell viability of the recombinant strain under different centrifugation conditions

Centrifugation is a common process of collecting cells, while the speed and time of centrifugation affect the harvest rate and viability of cells. Appropriate centrifugation conditions can improve the results. As shown in Fig. 2, the number of viable yeast cells varied greatly under different centrifugation conditions. At the same speed, the number of viable yeast cells showed a trend of increasing at first and then decreasing with the increase of time. When the centrifugation time was 15 min and the speed was 6000 rpm, the number of viable yeast cells reached up to 2.75 × 10^8^ CFU/mL. While the centrifugal speed was 7000 rpm, the number of viable yeast cells was at low levels. Consequently, the optimal centrifugation condition was 6000 rpm for 15 min.

### Optimization of protective compounds components and drying processes

**Fig. 3:**
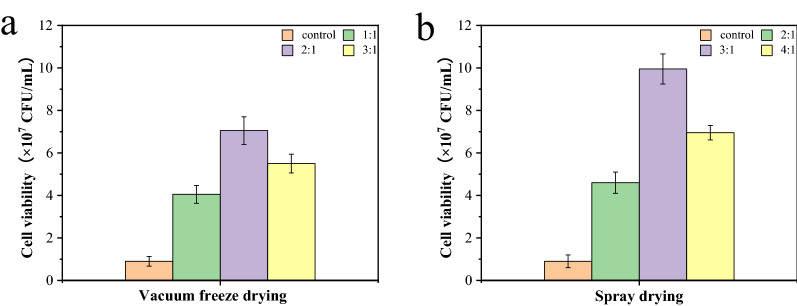
Effects of different ratios of protective compounds to yeast cells and drying process on *K. lactis* GG799(pKLAC1-ZPF1) agents. **a** was the cell viability of the agents obtained by vacuum freeze drying with the different ratios of protective compounds to yeast cells. **b** was the cell viability of the agents obtained by spray drying with the different ratios of protective compounds to yeast cells

In this work, for improving the activity of the yeast agents, the drying process of the agents was optimized. According to the above method, the *K. lactis* GG799(pKLAC1-ZPF1) agents were prepared by vacuum freeze drying and spray drying, respectively. As shown in Fig. 3, the protective compounds had significant effects on the viability of the yeast cells. When the ratio of protective compounds to yeast cells was 2:1 (w/w), the viability of the yeast cells was higher in vacuum freeze drying; while the ratio of protective compounds to yeast cells was 3:1 (w/w), the protective effect of spray drying was the best. Considering the production efficiency and the costs, the subsequent study was conducted using spray drying process, with a ratio of 3:1 (w/w) of the protective compounds to yeast cells.

**Fig. 4:**
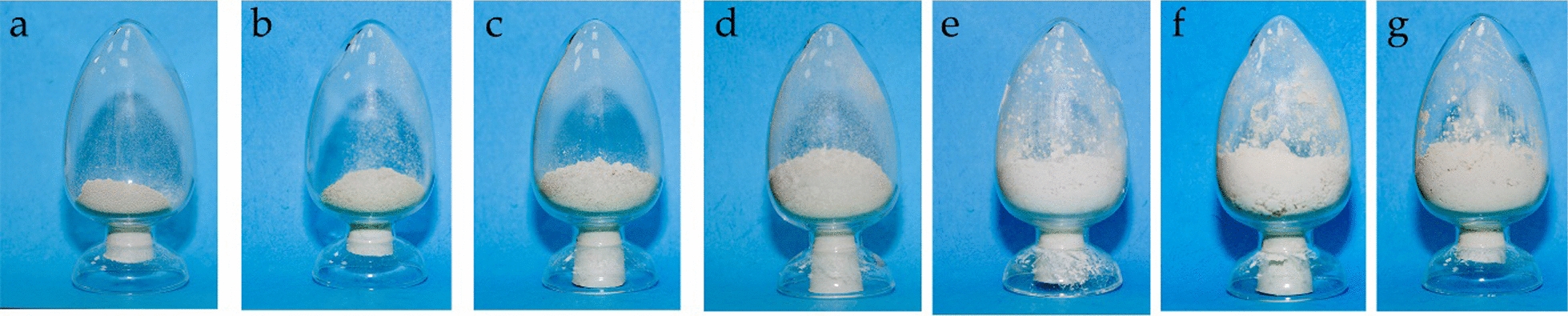
Products of *K. lactis* GG799(pKLAC1-ZPF1) agents with different ratios of protective compounds to yeast cells and different drying process. **a** agents without protective compounds by freeze drying (control); **b** agents prepared by freeze drying with protective compounds: yeast cells ratio at 1:1 (w/w); **c** agents prepared by freeze drying with protective compounds: yeast cells ratio at 2:1 (w/w); **d** agents prepared by freeze drying with protective compounds: yeast cells ratio at 3:1 (w/w); **e** agents prepared by spray drying with protective compounds: yeast cells ratio at 2:1; **f** agents prepared by spray drying with protective compounds: yeast cells ratio at 3:1; **g** agents prepared by spray drying with protective compounds: yeast cells ratio at 4:1

However, the yeast agents powders obtained by different drying processes showed different properties. The yeast agents powders obtained by spray drying were easy to be lumped, and the humidity and viscosity were larger, compared to the powders obtained by vacuum freeze drying (Fig. 4). Meanwhile, the addition of protective compounds also influenced the properties of the agents. The particle size and viscosity of the agents obtained with addition of protective compounds were lager than the agents obtained without addition of protective compounds.

### Cell viability after preservation

**Fig. 5:**
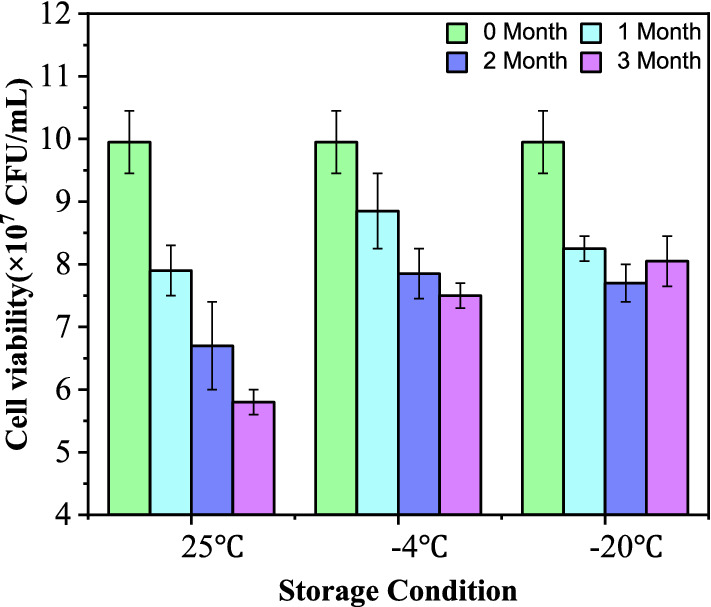
Effects of different storage conditions on cell viability of *K. lactis* GG799(pKLAC1-ZPF1) agents

The *K. lactis* GG799(pKLAC1-ZPF1) agents obtained by spray drying with the ratio of protective compounds to yeast cells at 3:1 (w/w) were placed at − 20 °C, − 4 °C and 25 °C, respectively. The viability of the yeast cells during storage was measured periodically. As shown in Fig. 5, the amount of active yeast in the *K. lactis* GG799(pKLAC1-ZPF1) agents gradually decreased with the storage time increased. The activity of yeast cells at − 20 °C was better than at − 4 °C and 25 °C. Meanwhile, the *K. lactis* GG799(pKLAC1-ZPF1) agents exhibited storage performance at − 20 °C and kept about 80.9% cell viability. As a result, the storage at − 20 °C is more favorable to maintain the activity and physiological status of the recombinant yeast agents.

### *K. lactis* GG799(pKLAC1-ZPF1) agents reactivation and mycotoxins degradation test

**Fig. 6:**
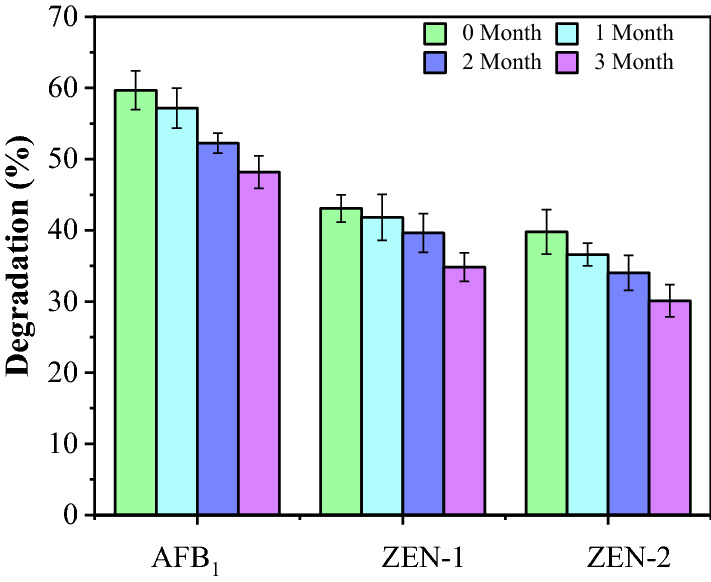
Degradation ratios of AFB1 and ZEN by the supernatants of reactivated yeast agents

The obtained *K. lactis* GG799(pKLAC1-ZPF1) agents were reactivated, and the fermentation supernatants were used to degrade AFB_1_ and ZEN. The degradation results of AFB_1_ and ZEN were shown in Fig. 6. It can be found that the yeast fermentation supernatants still exhibited degradation effect on AFB_1_ and ZEN after the reactivation, but the degradation ratios gradually decreased with the extension of storage time. After three months of storage, the degradation ratios were 48.2 ± 3.2% and 34.8 ± 2.8% for AFB_1_ and ZEN, respectively in reaction system 1; and 30.1 ± 2.7% for ZEN in reaction system 2. In system 2, the enzyme ZPF1 in the supernatants had no degradation effect on AFB_1_, due to the lack of malonic acid and H_2_O_2_.

To investigate the degradation effect of the fermentation supernatants on the real samples, degradation tests were performed on peanuts contaminated with AFB_1_ and ZEN. The degradation results were shown in Table 1. The supernatants of reactivated yeast agents degraded more than 80% of AFB_1_ and 55% of ZEN in the contaminated peanuts after twice treatments, with no significant differences (p > 0.05), which indicated that the recombinant strain *K. lactis* GG799(pKLAC1-ZPF1) was suitable for the degradation of AFB_1_ and ZEN in feed industry.

**Table 1:** Simultaneous degradation results of AFB_1_ and ZEN in contaminated peanuts by the supernatants of reactivated yeast agents

Mycotoxins	Mycotoxin concentration in samples (μg/kg)	Mycotoxin concentration detected in samples (μg/kg)	Mycotoxin concentration after once treatment (μg/kg)	Degradation ratio after once treatment (%)	Mycotoxin concentration after twice treatments (μg/kg)	Degradation ratio after twice treatments (%)
AFB_1_	100.0 500.0 2000.0	94.7 436.8 1785.4	54.7 243.7 957.0	42.2 ± 4.0 44.2 ± 1.6 46.4 ± 2.3	12.8 73.4 337.4	86.5 ± 0.9 83.2 ± 2.3 81.1 ± 3.7
ZEN	100.0 500.0 2000.0	96.2 476.7 1865.3	66.2 337.0 1330.0	31.2 ± 0.8 29.3 ± 3.6 28.7 ± 2.7	38.0 201.2 833.8	60.5 ± 1.4 57.8 ± 3.1 55.3 ± 2.4

Three parallel reactions were done for each group, and the standard deviations were listed

## Discussion

The cell viability of the *K.lacis* GG799(pKLAC1-ZPF1) agents during perparation process is mainly influenced by factors such as centrifugation conditions, protective compounds and drying processes.The number of viable yeast cells varies greatly under different centrifugation conditions. This was due to the fact that low speed reduced the number of viable yeast cells in the supernatants, while the shear force generated at high speed caused damage to the cells and lead to the death of the cells.

During the preparation of the *K. lactis* GG799(pKLAC1-ZPF1) agents, the ratio of protective compounds to yeast cells and the drying process had an impact on the viability of the yeast cells. When the dose of protective compounds was too little, the cells were exposed in large quantities during drying processing, which caused death of the cells and could not play the effective protective role; while the dose of protective compounds was too much, it affected the permeability of the cells, which resulted in the reduction in the viability of the yeast cells. Both of the parameters affect the viability of yeast cells (Palmfeldt et al. 2003).

There are many kinds of common protective compounds, involving sugars, proteins, polyhydroxy compounds, amino acids, etc. Meanwhile, the appropriate protective compounds and ratios vary from strain to strain. The protective compounds can not only avoid damage to the cells during drying processing, but also act as a support material during reversion and play a protective role (Coutinho et al. 1988; Berny and Hennebert 1991).

Vacuum freeze drying and spray drying appear to be convenient techniques for the dehydration. The former technology was commonly used as an effective process to produce active dry yeast. The powder obtained by vacuum freeze drying technology had the advantage of high viability and stability, easy for transportation and preservation, but the yield was low, and the production cost is relatively high (Stefanello et al. 2019). The spray drying technology was applicable for mass production; however, the inlet temperature would affect the viability of the yeast cells (Zhou et al. 2017). It is notable that the temperature during spray drying would greatly affect the property of the products. Too high temperature would inactivate the cells, while too low temperature was not beneficial to form the dried powder. Therefore, the import and export temperatures in spray drying processes need to be controlled in practical.

Active yeast agents should be stored at cool temperature and used as quickly as possible if yeast cell viability is to be maintained. As the cryopreservation conditions reduced the moisture content and oxygen pressure in the cells, resulting in slowing down the metabolism of the cells, leaving the cells in semidormancy or dormancy with the anti-aging effects. Even though the *K. lactis* GG799(pKLAC1-ZPF1) agents retained degradation efficiency after the reactivation, the long storage time decreased the activity of the agents, which affected the degradation effects of the fusion enzyme. Meanwhile, the possibility of practical application of this supernatants in the future food industry was verified.

In summary, the preparation process of the *K. lactis* GG799(pKLAC1-ZPF1) agents were developed and optimized. We investigated the viability of yeast cells for characterization of the effects on preparation processes for the *K. lactis* GG799(pKLAC1-ZPF1) agents. Based on the data obtained, the number of viable yeast cells varies greatly under different preparation conditions. It is obvious that optimal preparation process retains the maximum number of viable cells and reduces inactivity rate. Notably, the *K. lactis* GG799(pKLAC1-ZPF1) agents still showed excellent ability to degrade AFB_1_ and ZEN after 3 months of storage at − 20 °C and the supernatants of reactivated yeast agents were suitable for degradation of AFB_1_ and ZEN in practical samples. In addition, this work used the food-safe host *K. lactis* GG799 and the vector pKLAC1, so the fusion enzyme ZPF1 was secretory expressed in a food-grade manner, which property has advantages for applications in food or feed industries the future.

## Data Availability

Not applicable.
